# *PDP1* related ferroptosis risk signature indicates distinct immune microenvironment and prognosis of breast cancer patients

**DOI:** 10.3389/fphar.2025.1551325

**Published:** 2025-04-23

**Authors:** Yufeng Wang, Huifen Dang, Gongjian Zhu, Yingxia Tian

**Affiliations:** ^1^ Department of Breast Medical Oncology, Affiliated Cancer Hospital of Sun Yat-sen University, Gansu Hospital, Lanzhou, Gansu, China; ^2^ Department of Science and Education Section, Affiliated Cancer Hospital of Sun Yat-sen University, Gansu Hospital, Lanzhou, Gansu, China

**Keywords:** breast cancer, LASSO-cox regression analysis, prognosis, *PDP1*, *ACSL1*, *BNIP3*, *EMC2*

## Abstract

**Objective:**

We aim to construct a RiskScore model to aid in the early prognosis of breast cancer (BC).

**Methods:**

BC mRNA expression profiles were obtained from TCGA and GEO databases. Differential gene expression analysis identifies *PDP1*-ferroptosis-related genes. LASSO Cox regression was utilized to screen genes to build a RiskScore model, and survival analysis were performed to investigate the reliability in BC prognosis. Immune cell infiltration proportions were calculated using CIBERSORT and xCell algorithms. Single-cell data processing and analysis were conducted using “Seurat”, “monocle”, and “iTALK” packages. *PDP1* was silenced to validate its influence on the target genes.

**Results:**

Data from public databases revealed significant upregulation of *PDP1* in BC samples compared to normal tissues. A RiskScore model based on *PDP1*-related differential ferroptosis-related genes (FRGs) *ACSL1*, *BNIP3*, and *EMC2* was developed, which effectively predicted BC patient prognosis. High-risk BC samples exhibited poorer overall survival and were associated with immune microenvironment. The model remained significant in multivariate Cox regression analysis, indicating that it could independently predict the survival of BC patients. *ACSL1*, *BNIP3*, and *EMC2* were downregulated after knockdown of *PDP1*.

**Conclusion:**

RiskScore model constructed by *PDP1*-ferroptosis-related genes *ACSL1*, *BNIP3*, and *EMC2* is able to help predict the prognosis of BC patients.

## 1 Introduction

Breast cancer (BC) is a complex and heterogeneous disease with characteristic of the uncontrolled growth of abnormal cells in breast tissues (Feng et al., 2018). It is the most prevalent cancer among women all over the world and can affect men as well, albeit much less frequently (2023). BC may present in diverse manifestations, including ductal carcinoma *in situ* (DCIS) or invasive ductal carcinoma (IDC), each with distinct stages and grades (Makki, 2015). Risk factors for breast cancer include age, gender, family history, genetic mutations (such as BRCA1 and BRCA2), hormonal factors, and lifestyle choices (Sun et al., 2017). Early detection through regular mammograms, clinical breast exams, and breast self-exams is crucial for successful treatment (Kosters and Gotzsche, 2003). Treatment modalities may involve surgery, chemotherapy, radiation therapy, or targeted therapies, depending on the type and stage of the cancer (Debela et al., 2021). Ongoing research continues to advance our understanding of breast cancer biology, leading to improved diagnostic tools and therapeutic options aimed at enhancing patient outcomes and quality of life, where early diagnosis and prognosis prediction is the key.

*PDP1*, or Pyruvate Dehydrogenase Phosphatase Catalytic Subunit 1, is a gene that encodes the catalytic subunit of the phosphatase associated with the pyruvate dehydrogenase complex (PDC) (Alshamleh et al., 2023), which is a key enzyme involved in cellular energy metabolism, particularly in the conversion of pyruvate to acetyl-CoA, a crucial step in the tricarboxylic acid (TCA) cycle (Martin et al., 2005). In our unpublished research, *PDP1* has been detected to express significantly higher in BC compared to normal tissues (Wang et al., 2024), which is consistent with other research and lower *PDP1* expression in BC indicates better prognosis (Chen et al., 2022). In addition, PDP1 may promote BC progression by regulating the STAT3 signaling pathway (Wang et al., 2024).

Unlike classical apoptosis, autophagy, or necrosis, ferroptosis is a form of programmed cell death (PCD) with iron-dependent lipid peroxidation, resulting in the accumulation of toxic lipid hydroperoxides and eventual cell membrane damage (Li and Li, 2020; Li et al., 2020). Whether ferroptosis causes an oncogenic and tumor-suppressive event varies across different human cancers. Ferroptosis often dysfunctions in cancers and the utilization of ferroptosis inducers attracts much attention in cancer treatment (Chen et al., 2023). *Brucella abortus* rough-type mutant can induce ferroptosis in infected macrophages, and differentially expressed genes (DEGs) between this type and another type which cannot induce ferroptosis include *PDP1* (downregulated in the mutant) (Hu et al., 2023). Pyruvate metabolism and citric acid cycle patterns are able to help predict response to ferroptosis in gastric cancer, and *PDP1* is one of the regulators (Wang X. et al., 2022). Subtle relationships between *PDP1* and ferroptosis have been revealed in these researches. Here, we aim to utilize *PDP1*-related ferroptosis genes to help predict prognosis for BC which is valuable for intervention.

## 2 Materials and methods

### 2.1 Research object

In this study, mRNA expression profiles and corresponding clinical information of 1217 BC patients were obtained from The Cancer Genome Atlas (TCGA) database (https://tcga-data.nci.nih.gov/tcga/). This dataset comprised 1,104 breast cancer samples and 113 normal samples. After excluding samples with incomplete survival information, a total of 1,069 patients with comprehensive survival data were retained. Additionally, datasets from the Gene Expression Omnibus (GEO) database (https://www.ncbi.nlm.nih.gov/geo/) were collected, including GSE42568 (104 breast cancer samples, 17 normal tissue samples), GSE45827 (130 breast cancer samples, 11 normal tissue samples), GSE61594 (153 breast cancer samples, 11 normal tissue samples), GSE20685 (327 breast cancer samples), GSE21653 (266 breast cancer samples), GSE1456 (159 breast cancer samples), GSE173839 (breast cancer immunotherapy cohort), and GSE168410 (single-cell data of 12 breast cancer samples). GSE65194 contained 130 breast cancer samples, 11 normal breast tissue samples and 14 BRCA cell lines.

A set of 540 ferroptosis-related genes (FRGs), comprising drivers, markers, and suppressors, was sourced from the FerrDb database (http://www.zhounan.org/ferrdb) (Zhou and Bao, 2020) (Supplementary Table S1).

### 2.2 Differential gene expression analysis

All statistical analyses were performed using R language (4.3.1). Differential gene expression analysis was performed using the “limma” package (version 3.52.4) (Ritchie et al., 2015). Genes with an absolute log2 fold change (Log2 FC) greater than 0.5 and an adjusted p-value (p.adjust) less than 0.05 were considered differentially expressed.

### 2.3 Functional enrichment analysis

Functional enrichment analysis, including Gene Ontology (GO) categories (Biological Process (BP), Molecular Function (MF), and Cellular Component (CC)) and Kyoto Encyclopedia of Genes and Genomes (KEGG) Pathways, was conducted using the “clusterProfiler” package (version 4.7.1.2) (Yu et al., 2012). Enriched GO terms and KEGG pathways with a p-value less than 0.05 were considered statistically significant.

### 2.4 LASSO cox regression analysis

Univariate Cox regression analysis was applied to the FRGs, and genes significantly associated with breast cancer prognosis (p < 0.05) were selected as candidates. Subsequently, LASSO Cox regression analysis was performed using the “glmnet” package (version 4.1.7) (Friedman et al., 2010). The number of variables corresponding to the minimum λ value of the average error was selected to further screen the genes correlated with BC prognosis. The selected genes were then used to calculate the Risk Score for each sample using the formula provided:

$$RiskScore=\sum_{i=1}^{n}\text{Coe}f_{i}*X_{i}$$

where Coefi represented the risk coefficient for each factor calculated by the LASSO-Cox model, and Xi represented the gene expression values.

### 2.5 Survival analysis

Survival analysis was conducted using the “survival” package (version 3.5–5) and “survminer” package (version 0.4.9) based on the Kaplan-Meier (KM) method to estimate overall survival rates for different groups. The log-rank test was employed to assess the significance of differences in survival rates between different groups. The “timeROC” package (version 0.4) was utilized to generate ROC curves, and the area under the curve (AUC) values were calculated. A multivariate Cox regression model was employed to analyze whether the Risk Score could independently predict the survival of breast cancer patients in relation to other factors.

### 2.6 Calculation of immune cell infiltration proportions

The CIBERSORT software (Newman et al., 2015) was employed to calculate the relative proportions of 22 immune cell types within each sample. Using a gene expression matrix, CIBERSORT utilized a deconvolution algorithm with a predefined set of 547 barcode genes to characterize the composition of immune infiltrating cells. The sum of the estimated proportions of all immune cell types in each sample equaled 1. The single-sample Gene Set Enrichment Analysis (ssGSEA) algorithm was employed to calculate the abundance of 28 specific immune cell types. The xCell algorithm, implemented using the package “xCell” (https://github.com/dviraran/xCell), was used to compute the proportions of 64 immune cells in each sample.

### 2.7 Single-cell analysis

Single-cell data processing was performed using the “Seurat” package (version 4.3.0). Cells with less than 200 or greater than 2,500 features and greater than 5% mitochondrial counts were removed. The “NormalizedData” function was used for the standardization of single-cell RNA-sequencing (scRNA-seq) data. The “RunPCA” function was employed for principal component analysis (PCA). Unsupervised clustering of major cell subtypes was performed using the “FindClusters” function in “Seurat” package, followed by visualization using t-distributed stochastic neighbor embedding (t-SNE). Manual annotation was conducted using cell markers. Pseudotime analysis was carried out using the “monocle” package (version 2.26.0) (Qiu et al., 2017). The “iTALK” package (https://github.com/Coolgenome/iTALK, version 0.1.0) was utilized to explore cell-cell communication networks.

### 2.8 Cell culture

Two strains of BC cells, MCF-7 and MDA-MB-231, were purchased from National Collection of Authenticated Cell Cultures (Shanghai, China). The growth medium for MCF-7 and MDA-MB-231 consisted of Dulbecco’s Modified Eagle Medium (DMEM) (high glucose) (C0235, Grand Island Biological Company (GIBCO), Waltham, US) supplemented with 10% fetal bovine serum (FBS) (GIBCO, cat#C11995500BT) and 1% penicillin-streptomycin mixture (P1400, Beijing Solarbio Science & Technology Co., Ltd., Beijing, China). The cells were all cultured in a CO_2_-saturated incubator at 37°C with 5% CO_2_ and high humidity.

### 2.9 Transfection

Cells were observed under an inverted microscope to ensure they were in good condition with an appropriate density. The cells were processed in a super-clean hood using 0.25% trypsin-EDTA to generate a single-cell suspension. After centrifugation at 1,000 rpm for 5 min, the supernatant was removed, and the cells were resuspended in culture medium for counting. A 2 mL cell suspension was prepared in a 6-well plate. The culture plate was placed in a CO_2_ incubator and incubated for 24 h at 37°C. Subsequently, 1 μg of siRNAs was added, and cell transfection was carried out using Lipofectamine^TM^ 2000 (11668019, Invitrogen Corporation, Waltham, US). The sense and antisense sequences of siPDP1 were 5′-CCU​UGG​AUU​UGA​CAG​CAA​UTT-3′ and 3′-AUUGCUGUCAAAUCCAAGGDADT-5′, The relevant control siCtrl sense sequence was 5′-CAG​UAC​UUU​UGU​GUA​GUA​CAA​A-3′, and the antisense sequence was 3′-ACGUGACACGUUCGGAGAADTDT-5’.

### 2.10 QRT-PCR experiment

After collecting the transfected cell samples, Trizol (15596–018, Invitrogen) was used for lysis in RNA extraction. The samples were centrifuged at 2,000 rpm for 5 min, and the supernatant was removed. To the cell pellet, 1 mL of Trizol was added, thoroughly mixed, and left at room temperature for 5 min. The mixture was then transferred to new 1.5 mL centrifuge tubes. 200 μL of chloroform was added to each tube, and the tubes were inverted for 15 s. After standing at room temperature for 10 min, the tubes were centrifuged at 4°C and 12,000 rpm for 15 min. The upper liquid layer was moved into new 1.5 mL centrifuge tubes, an equal volume of pre-chilled isopropanol was added, and after thorough mixing, the mixture was allowed to stand at 4°C for 10 min. After centrifugation at 4°C and 12,000 rpm for 12 min, the supernatant was discarded. To the pellet, 1 mL of 75% ethanol (prepared freshly with DEPC water) was added for washing. After centrifugation at 4°C and 12,000 rpm for 5 min, most of the supernatant was removed. The pellet was further washed by repeating the centrifugation at 4°C and 12,000 rpm for 5 min, discarding the supernatant, and air-drying at room temperature. When the RNA pellet became nearly transparent, 50 μL of RNase-free water was added to completely dissolve it. The concentration and quality of the extracted RNA were determined using a spectrophotometer.

Following the instructions of the ReverTra Ace qPCR RT Kit (FSQ-101, Toyobo Co., Ltd., Osaka, Japan), 4 μL of 4× DNA Master Mix with gDNA Remover was taken. RNA template (0.8 μg) was added, and Nuclease-free Water was added to make up the volume to 16 μL. After gently mixing the reaction solution, it was incubated at 37°C for 5 min. Subsequently, 4 μL of 5× RTMasterMix II was added and mixed. The reaction proceeded at 37°C for 15 min, followed by 50°C for 5 min, and then 98°C for 5 min (20 μL reverse transcription system +360 μL ddH_2_O). The completed reaction mixture was stored at −20°C. Subsequently, a qRT-PCR experiment was conducted with the following reaction setup: TransStart Tip Green qPCR SuperMix (Transgen Biotech Co., LTD, Beijing, China) (10 μL), diluted cDNA (2 μL from a 20 μL reverse transcription system +360 μL ddH_2_O), primer F + R (Table 1) (2.4 μL), and water added to make up the volume to 20 μL. The PCR conditions were as follows: 95°C for 3 min, followed by 40 cycles of 95°C for 15 s, 60°C for 30 s, and 72°C for 30 s. The results were analyzed using the 7,500 Fast DX Real-time PCR instrument (Applied Biosystems, Waltham, US) and its associated software.

**TABLE 1:** Prime sequences for qRT-PCR.

Target gene	Primer sequence
ECM2-F	CTG​CTC​CGC​TAC​TGA​ACA​AAG​A
ECM2-R	TTC​GGT​CCA​TTC​CCA​CCT​G
BNIP3-F	GCC​ATC​GGA​TTG​GGG​ATC​TA
BNIP3-R	CCA​CCC​CAG​GAT​CTA​ACA​GC
ACSL1-F	GTG​GAA​CTA​CAG​GCA​ACC​CC
ACSL1-R	AGT​ATC​ATC​TGG​GCA​AGG​ATT​GA
GAPDH-F	ACA​ACT​TTG​GTA​TCG​TGG​AAG​G
GAPDH-R	GCC​ATC​ACG​CCA​CAG​TTT​C

### 2.11 Western blot (WB)

Proteins were extracted using Radio-Immunoprecipitation Assay (RIPA) buffer (P0013B, Beyotime Biotech Inc., Shanghai, China), separated with Sodium Dodecyl Sulfate Polyacrylamide Gel Electrophoresis (SDS-PAGE), and then transferred onto polyvinylidene fluoride (PVDF, ISEQ00010, Millipore Corporation, Bedford, US) membranes which were subsequently blocked with 5% non-fat milk to prevent non-specific binding. Subsequently, the membranes were incubated at 4°C for 12 h with primary antibodies: *BNIP3* monoclonal antibody (68091-1-Ig, 1:1,000, Proteintech Group, Inc., Rosemont, US), *ACSL1* polyclonal antibody (13989-1-AP, 1:1,000, Proteintech), *EMC2* polyclonal antibody (25443-1-AP, 1:1,000, Proteintech), *GAPDH* antibody (sc-47724, 1:1,000, Santa Cruz Biotechnology, Inc., Santa Cruz, US), and *PDP1* antibody (21176-1-AP, 1:1,000, Proteintech). Next, the membranes were incubated by secondary antibodies. Secondary antibody for *BNIP3* and *GAPDH* was Horseradish Peroxidase (HRP)-labeled Goat Anti-Mouse IgG (Heavy and Light chains (H + L)) (A0216, 1:5,000, Beyotime). Secondary antibody for *ACSL1*, *EMC2*, *PDP1* was HRP-conjugated Goat Anti-Rabbit (A0208, 1:5,000, Beyotime). Fluorescence imaging was performed using the ODYSSEY Clx imaging system (LI-COR, Inc., Lincoln, US).

### 2.12 Statistical analyses

Wilcoxon rank-sum tests were employed to compare gene expression differences and differences in immune cell infiltration between different groups. The Shapiro-Wilk test in R was used to test whether the sample conforms to a normal distribution. If the sample is found to conform to a normal distribution, use the “cor” function to conduct Pearson correlation analysis. Conversely, if the sample does not conform to a normal distribution, perform Spearman correlation analysis instead. In qRT-PCR experiment, comparisons between the sample means of the two groups were performed using the T-test. Differences were considered statistically significant when p < 0.05.

## 3 Results

### 3.1 Expression pattern, prognostic value and immune characteristics of PDP1 in BC

**FIGURE 1:**
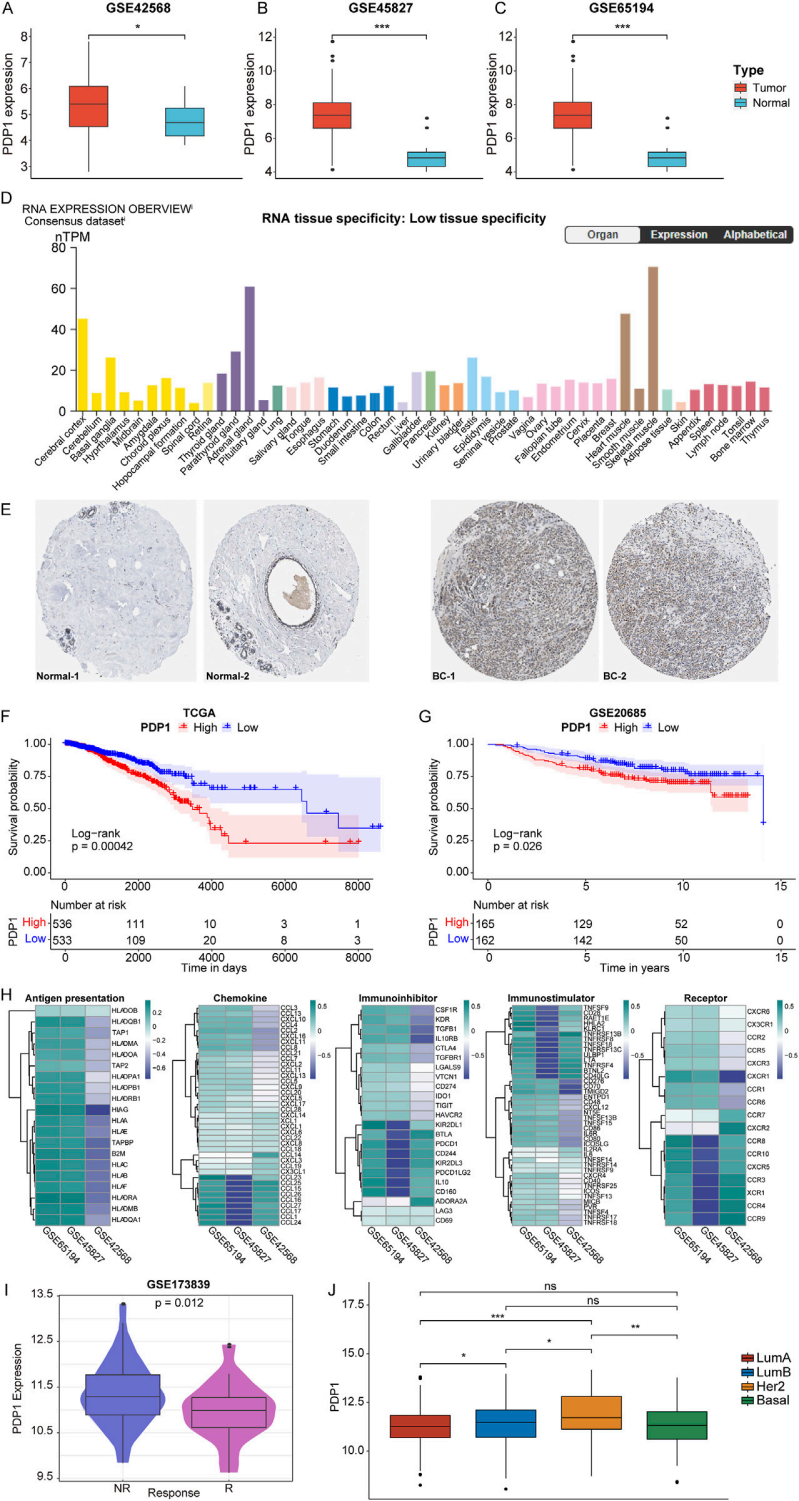
Expression pattern, prognostic value and immune signature results of *PDP1* in BC. **(A–C)**: Box plots of differential expression of *PDP1* in tumor and normal samples of GSE42568, GSE45827, and GSE61594 cohorts. **(D)**: *PDP1* expression levels in normal tissues of HPA database. **(E)**: Representative IHC of normal and tumor tissues in the HPA database. **(F, G)**: KM survival curves in the TCGA cohort and the GSE20685 cohort. P values are based on the log-rank test. **(H)**: Heatmap of correlation between *PDP1* and Antigen presentation, Immunoinhibitor, Immunostimulator, Receptor, and Chemokine. **(I)**: *PDP1* is differentially expressed in different states of breast cancer immunotherapy cohort patients. **(J)**: The expression of *PDP1* in different BC subtypes (*p < 0.05, **p < 0.01, ***p < 0.001).

In our unpublished previous study, *PDP1* has been found to be upregulated in breast cancer compared to normal tissues. By bioinformatics analysis, validation of *PDP1* expression in breast cancer samples *versus* normal samples was conducted using the GSE42568, GSE45827, and GSE61594 datasets. The results revealed a significant upregulation of *PDP1* expression in breast cancer samples across all three datasets (Figures 1A–C). Validation of *PDP1* expression using the Human Protein Atlas (HPA) database (https://www.proteinatlas.org/) demonstrated lower *PDP1* mRNA expression levels in normal tissues (Figure 1D). Additionally, in comparison to normal samples, breast cancer exhibited a relatively higher expression of *PDP1*, as illustrated by representative immunohistochemistry (IHC) images (Figure 1E).

Breast cancer samples from the TCGA (11.16113188) and GSE20685 (8.09563334299417) datasets were stratified into high (HPG) and low expression groups (LPG) based on the median expression values of the *PDP1*. Survival analysis of these two cohorts indicated that breast cancer samples with high expression of *PDP1* had poorer overall survival compared to those with low expression (Figures 1F, G). Furthermore, *PDP1* showed correlations with Antigen presentation, Immunoinhibitor, Immunostimulator, Receptor, and Chemokine (Figure 1H). In the GSE173839 dataset, *PDP1* was identified as a predictive factor for immunotherapy response in patients receiving immunotherapy (Figure 1I). Finally, we analyzed the expression of *PDP1* in different BC subtypes and discovered that *PDP1* was highly expressed in Her2 subtype (Figure 1J).

### 3.2 PDP1-related differential FRGs and their functional information in BC

**FIGURE 2:**
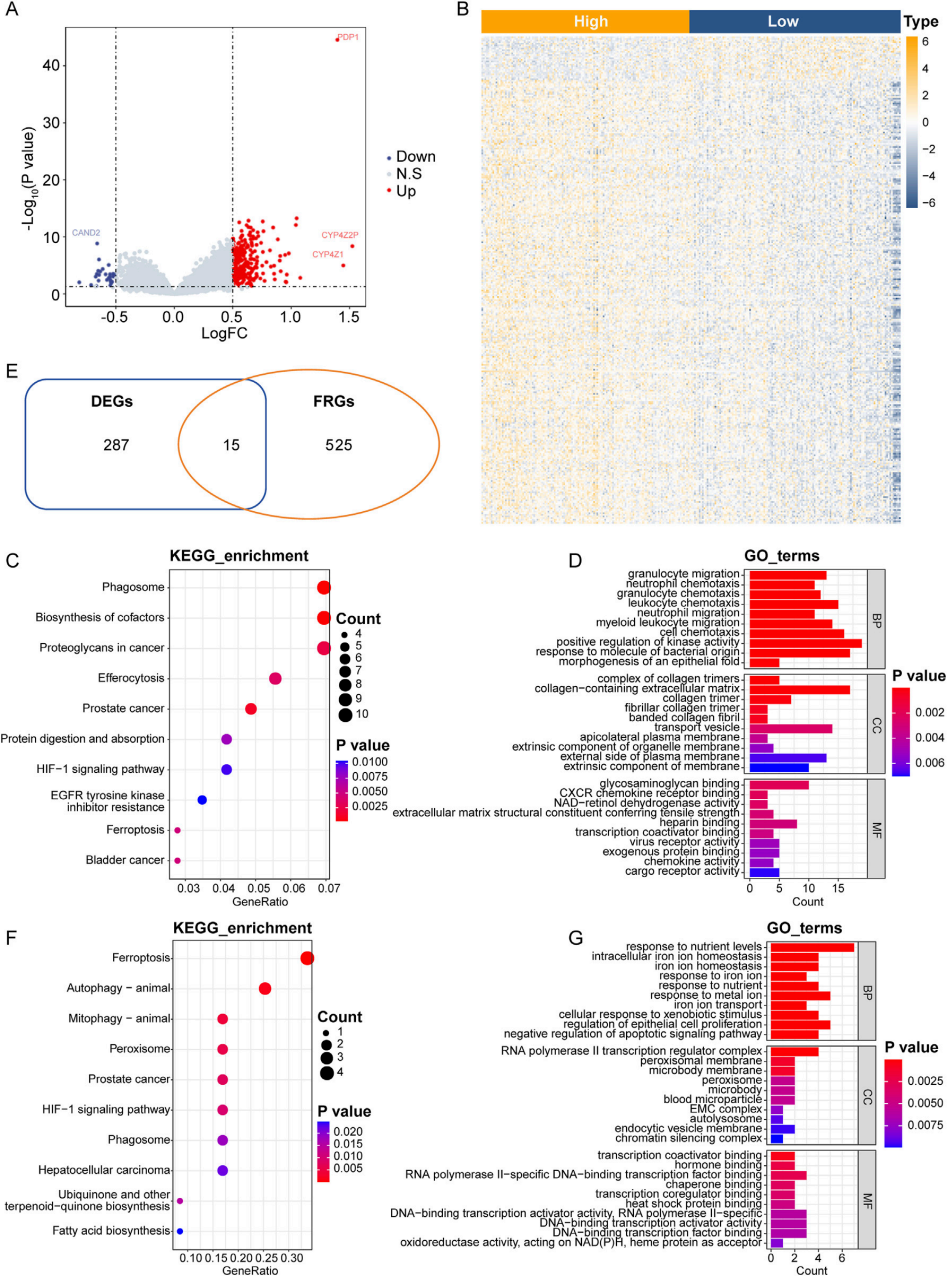
*PDP1*-related differential FRGs and functional enrichment analysis in BC. **(A)**: Volcano plot of differential analysis of the GSE21653 cohort. **(B)**: Heatmap of DEGs in the GSE21653 cohort. **(C)**: Dot plot of the top 10 most significantly enriched KEGG pathways. **(D)**: Histogram of the top 10 significantly enriched BP, CC and MF. **(E)**: Intersection venn Hdiagram of DEGs and FRGs. **(F, G)**: Top 10 significantly enriched KEGG pathways and the top 10 significantly enriched GO pathways.

To gain insights into the biological processes, molecular functions, and potential pathways associated with *PDP1* and its related ferroptosis genes in BC, we performed functional enrichment analysis. Breast cancer samples from the GSE21653 dataset were divided into HPG and LPG based on the median expression values of the *PDP1* (6.49). Differential gene expression analysis was then conducted between HPG and LPG. HPG exhibited a total of 302 DEGs, including 275 upregulated genes and 27 downregulated genes compared to LPG (Figures 2A, B).

Functional enrichment analysis, including GO and KEGG pathways, was performed on the 302 DEGs. Among the significantly enriched KEGG pathways (31 pathways with p-value <0.05), ferroptosis pathway was included. Additionally, there were 623 significantly enriched BP terms, 51 MF terms, and 55 CC terms. The top 10 significantly enriched KEGG pathways and the top 10 enriched GO pathways were shown in Figures 2C, D. Detailed results of the enrichment analysis could be found in Supplementary Table S2.

The intersection of the 302 DEGs and the 540 FRGs resulted in 15 genes, which were considered as differentially expressed FRGs (Figure 2E; Supplementary Table S3).

Further GO and KEGG enrichment analysis was conducted on the 15 differentially expressed FRGs associated with *PDP1*. Thirteen KEGG pathways, including the ferroptosis pathway, were significantly enriched, along with 552 BP terms, 41 MF terms, and 32 CC terms. The top 10 significantly enriched KEGG pathways and the top 10 enriched GO pathways were presented in Figures 2F, G. Detailed results of the enrichment analysis could be found in Supplementary Table S4.

### 3.3 RiskScore model based on PDP1-related differential FRGs could predict BC patient prognosis

**FIGURE 3:**
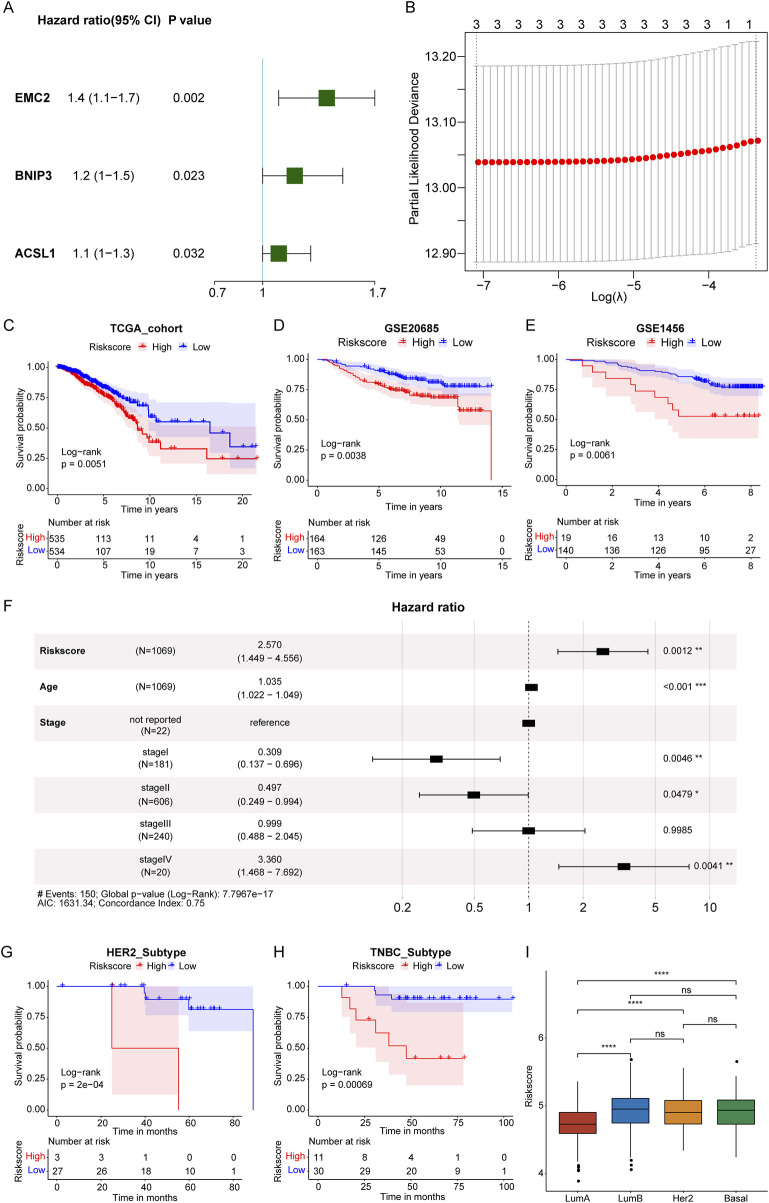
Construction of RiskScore model. **(A)**: Forest plot of univariate Cox analysis of 15 *PDP1*-related differential FRGs genes. **(B)**: Determining the optimal lambda in the LASSO regression model. **(C–E)**: KM survival curves in the TCGA and GEO cohorts. **(F)**: Multivariate Cox regression analysis forest plot. **(G, H)**: KM survival curves of HER2-positive subtypes and TNBC subtypes. **(I)**: Risk score in Lumb, Her2, Basal, and LumA subtypes. (****p < 0.0001, ns represents non-significance).

By harnessing the power of molecular data and bioinformatics, we aim to provide clinicians with a robust and quantitative approach to assess the prognosis of breast cancer patients, and the construction of a RiskScore model will be helpful. In breast cancer patients from the TCGA-BRCA dataset with complete clinical survival information, a univariate Cox regression analysis was conducted using the expression values of 15 *PDP1*-related differentially expressed FRGs as continuous variables. Hazard ratios (HR) were calculated for each gene, and three genes with a significant p-value (<0.05) were identified (Figure 3A).

The three selected genes underwent LASSO Cox regression analysis, and the optimal number of genes was determined to be three based on the lambda values corresponding to different numbers of genes in the LASSO Cox analysis (Figure 3B, where the lambda value was minimized). The selected genes were *EMC2* (Endoplasmic Reticulum Membrane Protein Complex Subunit 2), *BNIP3* (BCL2 and adenovirus E1B 19-kDa-interacting protein 3), and *ACSL1* (Acyl-CoA Synthetase Long-chain Family Member 1). It has been showed that these three genes were closely associated with ferroptosis (Zhou and Bao, 2020). Thus, *EMC2*, *BNIP3*, and *ACSL1* were selected for further analysis.

Subsequently, a RiskScore model predicting patient survival was established by weighting the gene expression levels with the coefficients obtained from the LASSO Cox regression analysis. The formula was as follows: RiskScore = 0.25093327 × *EMC2* expression value +0.08844663 × *BNIP3* expression value +0.07694525 × *ACSL1* expression value. RiskScores were calculated for each patient, and based on the median RiskScore, breast cancer samples in the training set of the TCGA cohort and the validation set of GSE20685 were stratified into high (HRSG) and low-risk groups (LRSG). Survival analysis revealed that HRSG samples had poorer overall survival in both the training and validation sets (Figures 3C, D). Additionally, samples in GSE1456 were divided into two groups based on the optimal cutoff value for RiskScores, showing that high-risk breast cancer samples had poorer overall survival compared to low-risk samples (Figure 3E).

Furthermore, time-dependent ROC analysis demonstrated that the AUC values for the 1-year, 3-year, and 5-year survival periods in the training set (TCGA-BRCA) were 0.56, 0.58, and 0.56, respectively (Supplementary Figure S1A). In the validation set (GSE20685), the AUC values for the 1-year, 3-year, and 5-year survival periods were 0.71, 0.69, and 0.65, respectively (Supplementary Figure S1B). For GSE1456, the AUC values for the 1-year, 3-year, and 5-year survival periods were 0.78, 0.59, and 0.60, respectively (Supplementary Figure S1C). These results indicated that the model based on the TCGA-BRCA training set was effective in predicting the prognosis of breast cancer patients.

In a multivariate Cox regression analysis with factors of age, stage, and RiskScore, it was determined whether the RiskScore was an independent prognostic indicator (Figure 3F). The results indicated that both RiskScore and age remained significantly associated with overall survival, with higher risk scores indicating a greater risk of death and serving as adverse prognostic factors (HR = 2.570, 95% CI: 1.449–4.556, P = 0.0012).

In the GSE61594 dataset, survival analysis of the risk score in different breast cancer subtypes revealed that in Human Epidermal Growth Factor Receptor 2 (HER2)-positive and Triple-Negative Breast Cancer (TNBC) subtypes, high-risk breast cancer samples had poorer overall survival compared to low-risk samples (Figures 3G, H). Moreover, we analyzed Risk score in different BC subtypes and found that the Risk score was significantly higher in Lumb, Her2, and Basal subtypes than LumA subtype (Figure 3I).

### 3.4 Differential immune characteristics in BC patients with high and low risk scores

**FIGURE 4:**
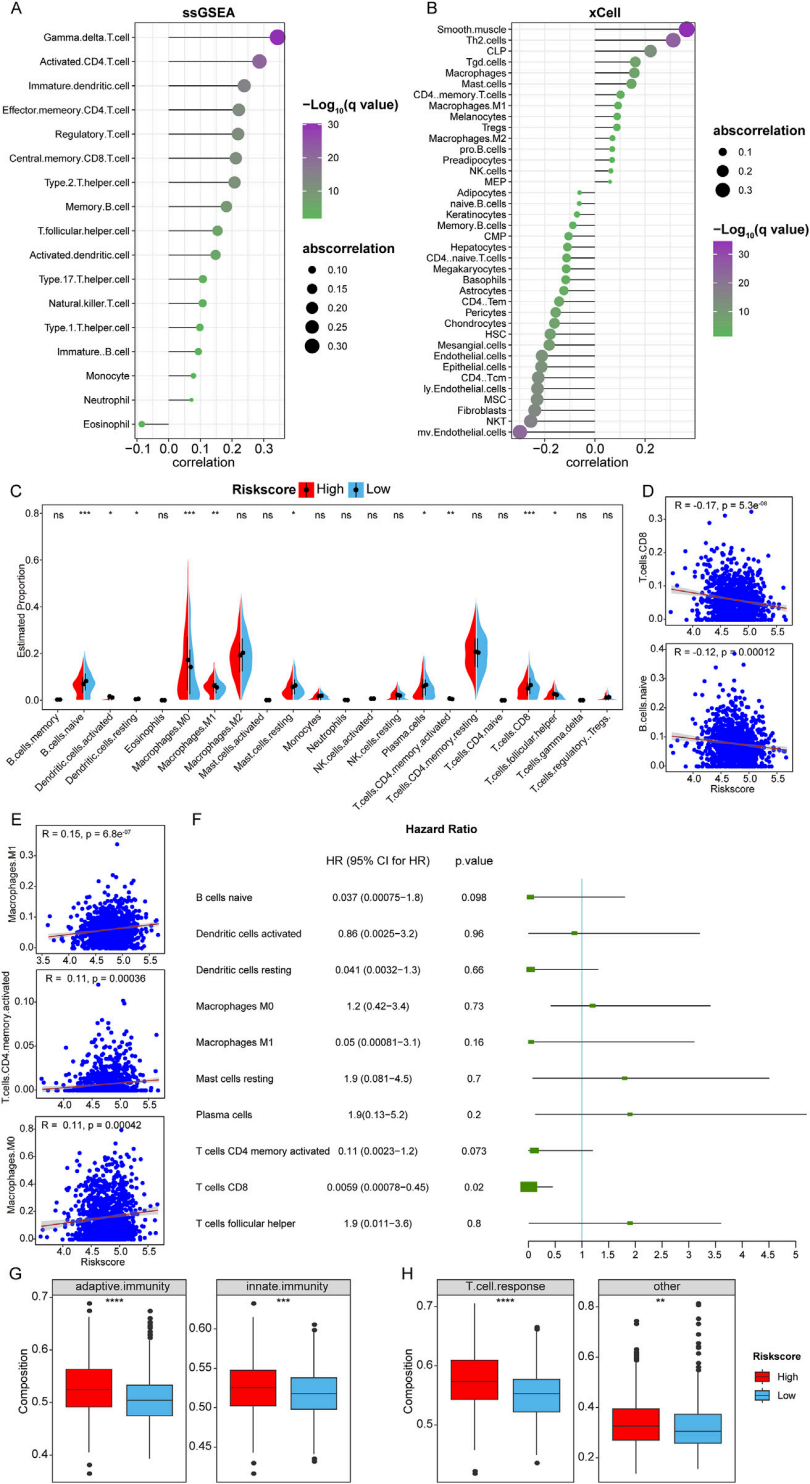
Immune infiltration analysis. **(A, B)**: Correlation between RiskScore and infiltration proportion of immune infiltrating cells in ssGSEA and xCell algorithm. **(C)**: Box plot of the difference in immune cell infiltration between 22 types of immune infiltrating cells between samples in HRSG and LRSG. (*p < 0.05, **p < 0.01, ***p < 0.001, ****p < 0.0001, ns represents non-significance). **(D, E)**: Scatter plots of correlation between RiskScore and significantly different immune infiltrating cells. **(F)**: univariate Cox analysis. **(G)**: Differences in adaptive immunity and innate immunity between HRSG and LRSG. **(H)**: Differences in T cell responses between HRSG and LRSG.

Immune characteristics can provide insights into the tumor microenvironment and the interactions between cancer cells and the immune system. Using the TCGA-BRCA dataset, the correlation between RiskScore and the abundance of various immune-infiltrating cells was analyzed using ssGSEA and the xCell algorithm. In ssGSEA, the RiskScore demonstrated a significant positive correlation with 16 immune cells, including Gamma delta T cells, Activated CD4 T cells, and Immature dendritic cells. Conversely, there was a significant negative correlation between RiskScore and Eosinophils (Figure 4A). The xCell analysis revealed a significant negative correlation between RiskScore and 23 immune cells, such as microvascular Endothelial cells (mv.Endothelial.cells), Natural Killer T cells (NKT), and Fibroblasts. Additionally, RiskScore exhibited a positive correlation with 15 immune cell types, including Common Lymphoid Progenitors (CLP), T-helper two cells (Th2. cells), and Smooth Muscle cells (Figure 4B).

Additionally, the CIBERSORT algorithm was used to calculate the relative abundance of 22 immune-infiltrating cells in breast cancer samples from the TCGA-BRCA cohort. Stratifying the samples into HRSG and LRSG based on the median of RiskScore, the analysis revealed significant differences in the infiltration of 10 immune cells between HRSG and LRSG, among them, naive B cells, resting dendritic cells, resting mast cells, plasma cells, CD8 T cells were significantly higher in LRSG, and activated dendritic cells, M0 Macrophages, M1 Macrophages, activated memory CD4 T cells, and follicular helper T cells were significantly higher in HRSG (Figure 4C). Further analysis of the Pearson correlation between RiskScore and significantly different immune-infiltrating cells showed a significant negative correlation with CD8 T cells and naive B cells, and a significant positive correlation with M1 Macrophages, activated CD4 Memory T cells, and M0 Macrophages (Figures 4D, E). Furthermore, univariate Cox analysis showed that CD8 T cells were correlated with prognosis of patients with breast cancer (Figure 4F), indicating that CD8 T cells might predict the prognosis of BC.

Furthermore, it was observed that adaptive immunity and innate immunity showed relatively stronger in HRSG than LRSG (Figure 4G). Tumors with high RiskScore were associated with a relatively stronger T-cell response (Figure 4H).

### 3.5 RiskScore in BC samples at single-cell level

**FIGURE 5:**
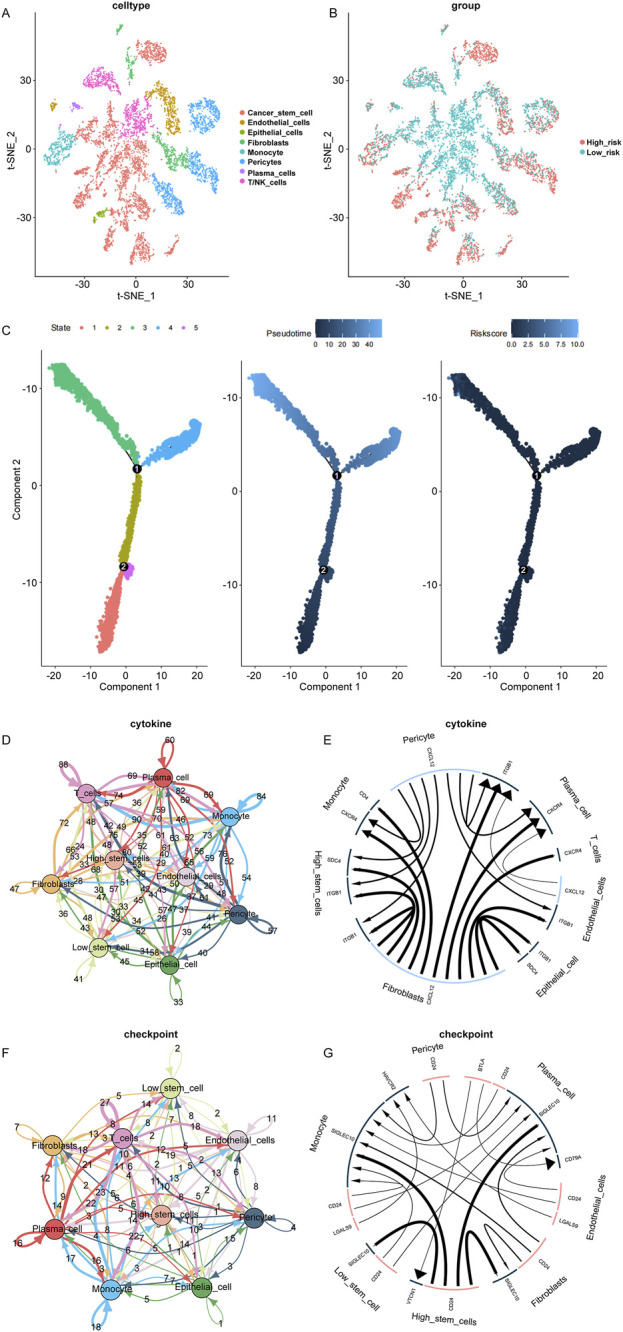
Single-cell data analysis. **(A)**: Cell clusters identified based on single-cell data using the t-SNE algorithm, with a total of 25 clusters. **(B)**: Classification results of cell clusters based on t-SNE algorithm. **(C)**: Pseudotime analysis of breast cancer cells in different cell states (left). Pseudotime pattern of pseudotime analysis of breast cancer cells (middle). RiskScore of pseudotime analysis of breast cancer cells (right). **(D, E)**: Cytokine cell-cell communication network. **(F, G)**: Checkpoints cell-cell communication network.

Investigating breast cancer at the single-cell level is able to provide an understanding of cellular heterogeneity. Using the GSE168410 scRNA-seq dataset, a single-cell gene expression profile was obtained, and the data were processed and filtered for subsequent analysis. PCA analysis was performed to reduce dimensionality using 5,000 variable genes, and 25 cell clusters were identified using “Seurat” (Supplementary Figure S2). The cell annotation results were depicted in Figure 5A. The levels of RiskScore in the identified cells were shown in Figure 5B. Pseudotime analysis was conducted on breast cancer cells, defining 5 cell states (Figure 5C left). As pseudotime passed (Figure 5C center), RiskScore of breast cancer cells tended to increase (Figure 5C right).

The “iTALK” tool was utilized to analyze the cell-cell communication network between RiskScore subgroups, including cytokines and checkpoints. In the cytokine module, SDC4 and ITGB1 were identified as more active signaling pathways in breast cancer with high RiskScore (Figures 5D, E). In the checkpoint module, CD24 and VTCN1 were recognized as more active signaling pathways related to immune escape in breast cancer with high RiskScore (Figures 5F, G).

### 3.6 Expression of key genes in the RiskScore model in BC

**FIGURE 6:**
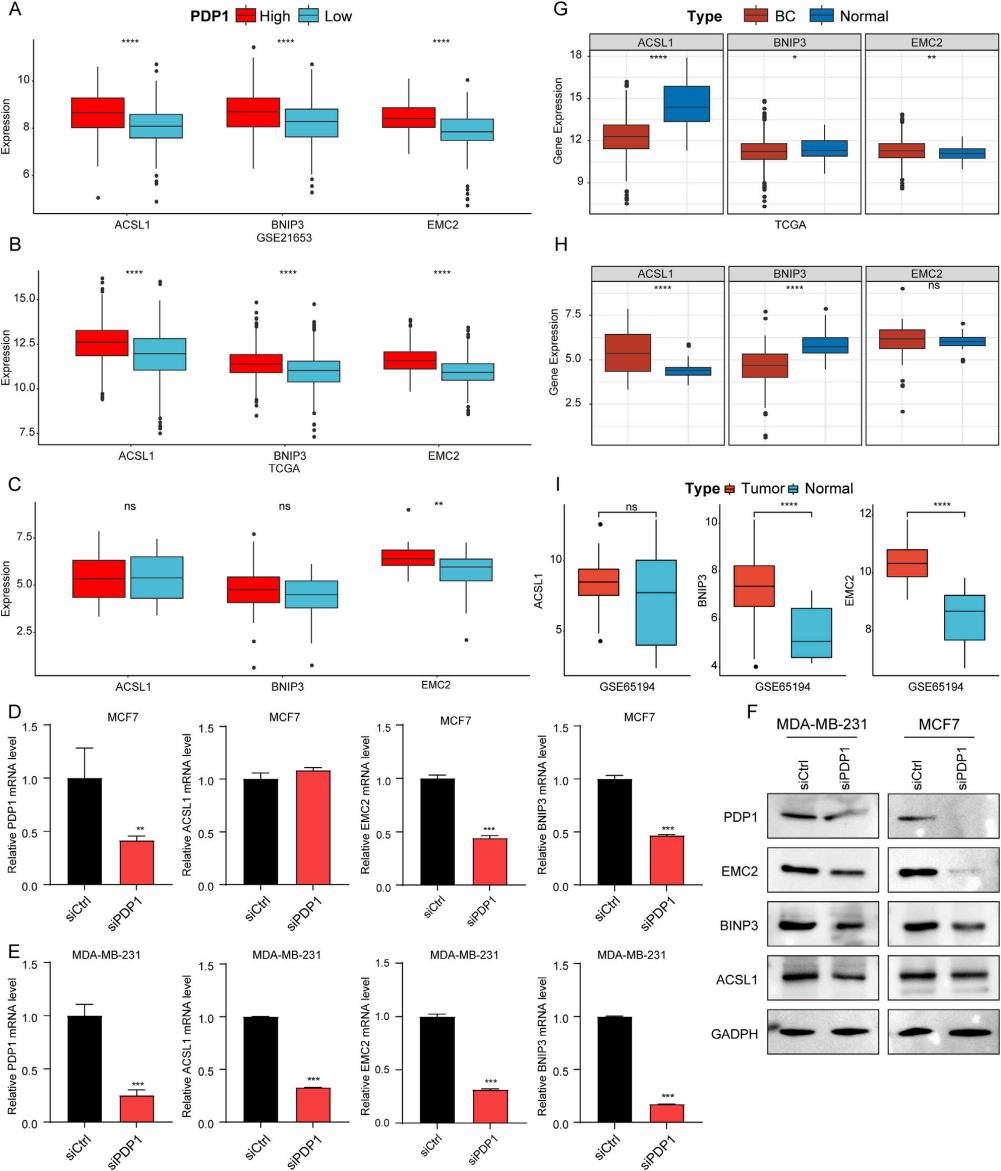
Validation of the expression of genes using in the RiskScore model. **(A–C)**: Box plots of differential expression of *ACSL1*, *BNIP3*, and *EMC2* in samples from HPG and LPG in GSE21653, TCGA cohort and CCLE database. **(D, E)**: Expression levels detected by qRT-PCR of *ACSL1*, *BNIP3*, and *EMC2* after *PDP1* knockdown in MCF7 and MDA-MB-231 cell line, respectively. **(F)**: Protein levels detected by WB of *ACSL1*, *BNIP3*, and *EMC2* after *PDP1* knockdown in MCF7 and MDA-MB-231 cell line, respectively. **(G–I)**: Differential expression of *ACSL1*, *BNIP3*, and *EMC2* in BC and normal samples in TCGA cohort, CCLE database, and GSE42568 dataset.

Besides constructing the RiskScore model, we also explored the expression of genes used in the model. The expression patterns of the three genes (*EMC2*, *BNIP3*, and *ACSL1*) were analyzed in HPG and LPG using the GSE21653 and TCGA datasets. The results showed that, compared to LPG, *EMC2*, *BNIP3*, and *ACSL1* were all significantly upregulated in HPG samples in both datasets (Figures 6A, B). Validation in the Cancer Cell Line Encyclopedia (CCLE) database confirmed that the expression of *EMC2* was significantly higher in HPG cell lines compared to LPG cell lines (Figure 6C). Furthermore, we validated *PDP1*’s influence on *EMC2*, *BNIP3*, and *ACSL1*, showing that after silencing *PDP1*, all three genes were downregulated (Figures 6D, E), and the influence was also validated by WB (Figure 6F). The grouping of blots was cropped from different gels, and the original images were attached in Supplementary Figure S3.

Furthermore, the expression of these three genes in BC samples *versus* normal samples was analyzed. In TCGA dataset, compared to normal samples, *ACSL1* and *BNIP3* were significantly downregulated, while *EMC2* was significantly upregulated in BC samples (Figure 6G). In the CCLE database, *ACSL1* expression was significantly higher in BC cell lines than in normal cell lines, and *BNIP3* expression was significantly lower in BC cell lines than in normal cell lines (Figure 6H), consistent with the TCGA data. Subsequently, further validation of gene expression was conducted in GSE65194 to analyze the expression patterns of the three genes in BC samples *versus* normal samples. The results showed that, relative to normal samples, *BNIP3*, and *EMC2* were significantly upregulated in BC samples (Figure 6I). Our data indicated that *BNIP3* consistently displayed significantly lower expression levels in breast cancer samples across various datasets, warranting further investigation.

## 4 Discussion

In our research, functional enrichment analysis indicated the relationship between *PDP1* and ferroptosis in BC. Then we focused on 15 *PDP1*-related differential FRGs and three genes *ACSL1*, *BNIP3*, and *EMC2* were selected to build an effective RiskScore model to predict BC prognosis. The RiskScore was related to immune microenvironment.

Cancer cells (including BC) exhibit enhanced glycolysis and diminished mitochondrial oxidative phosphorylation, and this metabolic shift may be partly attributed to the inhibition of PDC activity. *PDP1* primarily activates PDC by removing phosphate groups from PDC, thereby influencing cellular metabolism (Saunier et al., 2016). Alterations in cellular metabolism may indirectly influence the sensitivity to ferroptosis. In acute myeloid leukemia (AML), the increase d expression of PDP1 is associated with metabolic reprogramming following FLT3 inhibition (Alshamleh et al., 2023). PDP1 facilitates survival in the context of FLT3 by promoting oxidative phosphorylation (OXPHOS) metabolism (Alshamleh et al., 2023). This metabolic reprogramming may indirectly influence the sensitivity of cells to ferroptosis. Thus, *PDP1* may affect the sensitivity of tumor cells to iron deposition by changing cell metabolism. PDP1 is highly expressed in a variety of cancers, usually related to bad prognosis, such as colorectal cancer (Yuan et al., 2024) and BC (Wang et al., 2024). By dividing BC samples into HPG and LPG, we identified 15 *PDP1*-related differential FRGs, among which *ACSL1*, *BNIP3*, and *EMC2* were screened to construct the RiskScore model. The three genes all expressed significantly higher in HPG. Knockdown experiment validated that silence of *PDP1* could downregulate the expression levels of the three genes. Interestingly, in BC samples and normal samples, the expression patterns of these FRGs were not always the same in different datasets possibly due to the double-edged effect of ferroptosis in BC (Dang et al., 2022). The expression levels of ACSL vary in different cancer and subtypes. *ACSL1* exhibits elevated expression levels in hepatocellular carcinoma, BC, ovarian cancer, and colorectal cancer, while displays low expression in esophageal adenocarcinoma and renal cell carcinoma (Zhang and Wang, 2023). *ACSL1* is linked to poorer prognosis in BC but better prognosis in lung cancer (Chen et al., 2016). *ACSL1* can mediate ferroptosis caused by conjugated linoleate α-eleostearic acid (αESA), which is able to inhibit tumorigenesis and metastasis in murine BC *in vitro* (Beatty et al., 2021). In our study, *BNIP3* expressed significantly lower in BC compared to normal tissues in three datasets. *BNIP3* protein has been reported to suppress tumorigenesis in mouse model by overproduction of reactive oxygen species (ROS) generated by dysfunctional mitochondria (Chourasia et al., 2015). However, under hypoxic conditions, *BNIP3* expresses higher in BC and activate autophagy, further activate malignant phenotypes of BC (Zhang et al., 2022). The relationship of ferroptosis and *BNIP3* is linked by hypoxia, because ferroptosis is a kind of oxidative damage-related cell death and *BNIP3* is gene related to hypoxia (Zheng et al., 2023). *BNIP3* has also been applied in a RiskScore model of cholangiocarcinoma, expressing significantly lower in cholangiocarcinoma than normal tissues but higher expression is related to poorer prognosis (Wang Z. et al., 2022), as in BC. The collapse of EMC, namely, endoplasmic reticulum membrane protein complex, has broad implications for various cellular processes, such as organelle communication and lipid homeostasis which are related to ferroptosis, and tumors. In BC, increased expression of *EMC2* facilitated by non-coding RNAs (ncRNAs) is associated with an unfavorable prognosis (Liu et al., 2021). *EMC2* has been identified as a predictive gene for esophageal cancer prognosis where higher expression indicates poorer prognosis as well (Zhu et al., 2021).

RiskScore was found to be related to immune landscape in BC by all algorithms of ssGSEA, xCell, and Cybersort. RiskScore had a significant positive correlation with M1 Macrophages which was probably caused by *ACSL1*. Exposure to palmitate led to the development of a foamy and inflammatory phenotype in macrophages, accompanied by an elevation in *ACSL1* expression. Inhibiting or knocking down *ACSL1* mitigated macrophage foaming and inflammation triggered by palmitate stimulation, achieved through the downregulation of FABP4 expression. *ACSL1* serves as a pivotal regulator in the inflammatory response and macrophage foaming induced by short-term palmitate exposure or acute high-fat feeding (Al-Rashed et al., 2023). In cell renal cell carcinoma, the subgroup characterized by elevated *ACSL1* expression showed enrichment in pathways associated with fatty acid metabolism and demonstrated heightened expression of genes linked to ferroptosis. Meanwhile, the subgroup with low *ACSL1* expression displayed elevated immune and microenvironment scores (Yang et al., 2023). In BC, there was a positive correlation between the expression of *BNIP3* and the levels of immune and stromal cells (Yu et al., 2023). By single-cell analysis of various epithelial cancers, the predominant expression of *BNIP3* occurs in epithelial cells within the tumor microenvironment, rather than in immune cells (Zhu et al., 2022). The influence of FRGs on tumor microenvironment is complicated in various cancers even in the same cancer, and much more research is required in this area.

Some limitations in this study should be equally noted. Firstly, this study used retrospective data from public databases, therefore the prediction model needs to be further verified in large independent clinical cohort. Secondly, the specific contributions of *ACSL1*, *BNIP3* and *EMC2* to the model need to be investigated using ablation experiments. Finally, the regulatory mechanisms of *PDP1* on *ACSL1*, *BNIP3* and *EMC2* need to be further explored *in vivo* and *in vitro* experiments.

## 5 Conclusion

To summarize, we validated the expression of *PDP1* in BC at first and found the relationship between ferroptosis and *PDP1*. Then we identified 15 *PDP1*-related differential FRGs and selected three of them (*ACSL1*, *BNIP3*, and *EMC2*) by LASSO Cox regression analysis. A RiskScore model was constructed by the three genes and it was shown to be able to predict prognosis of BC patients. A significant relation was detected between the RiskScore and immune cells. Our risk scoring model can layered breast cancer patients to help doctors identify patients with poor prognosis, thereby formulating more active treatment plans for high -risk patients.

## Data Availability

Publicly available datasets were analyzed in this study. This data can be found at the NCBI repository, accession numbers: GSE42568, GSE45827, GSE61594, GSE20685, GSE21653, GSE1456, GSE173839, GSE168410 and GSE65194.

## Supplementary material

SUPPLEMENTARY FIGURE S1 ROC curves of the training set **(A)** and the validation sets **(B, C)**.

SUPPLEMENTARY FIGURE S2 25 cell clusters identified in single-cell analysis.

SUPPLEMENTARY FIGURE S3 Original WB images based on MCF7 and MDA-MB-231 cell lines.
