# Supplementary figures and images for: PDP1 related ferroptosis risk signature indicates distinct immune microenvironment and prognosis of breast cancer patients
Source: Front Pharmacol. 2025 Apr 23;16:1551325. doi: 10.3389/fphar.2025.1551325 (PMC12055530; doi:10.3389/fphar.2025.1551325)

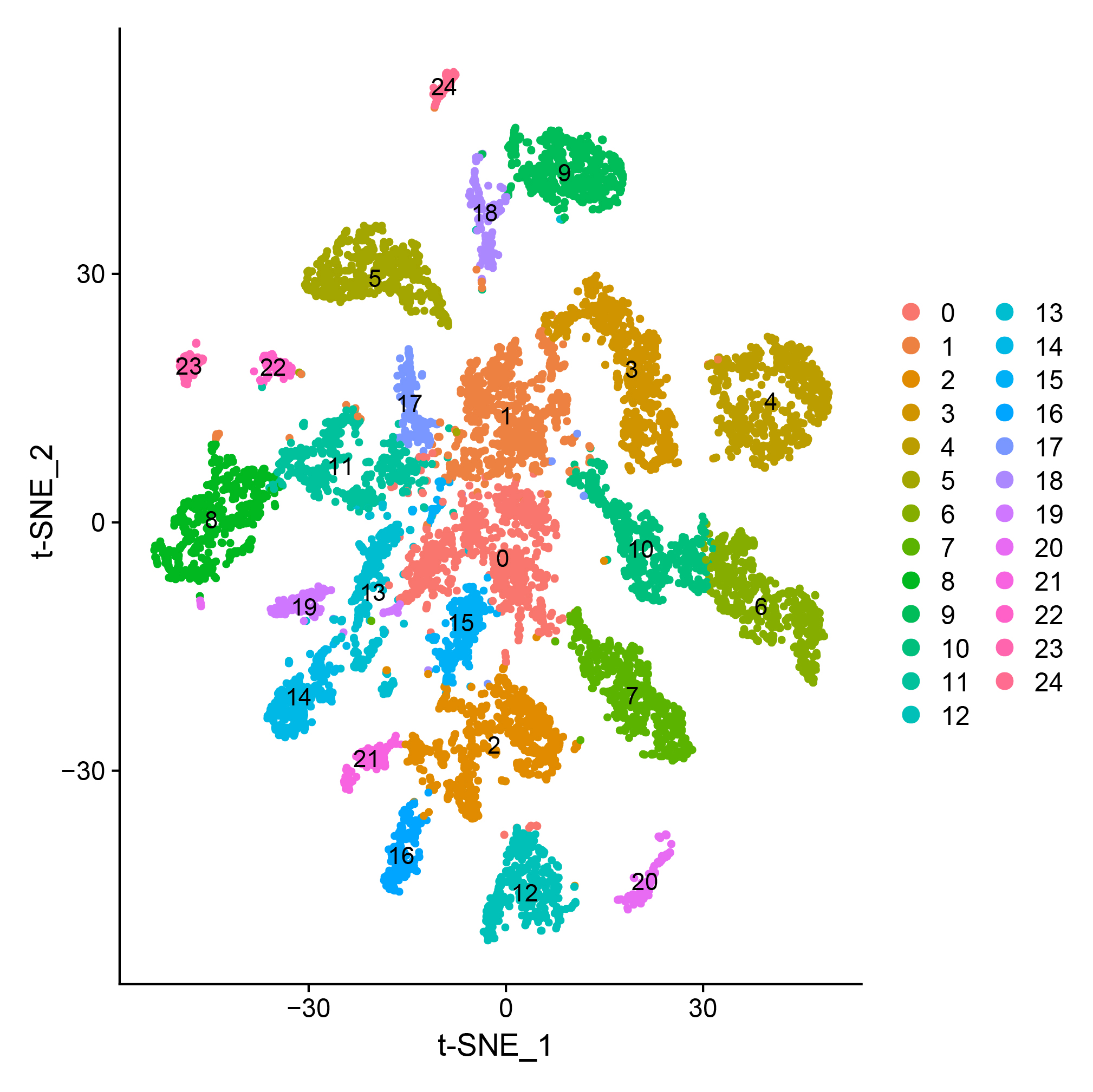

Supplement: Supplementary file 2 [file Image2.jpeg]

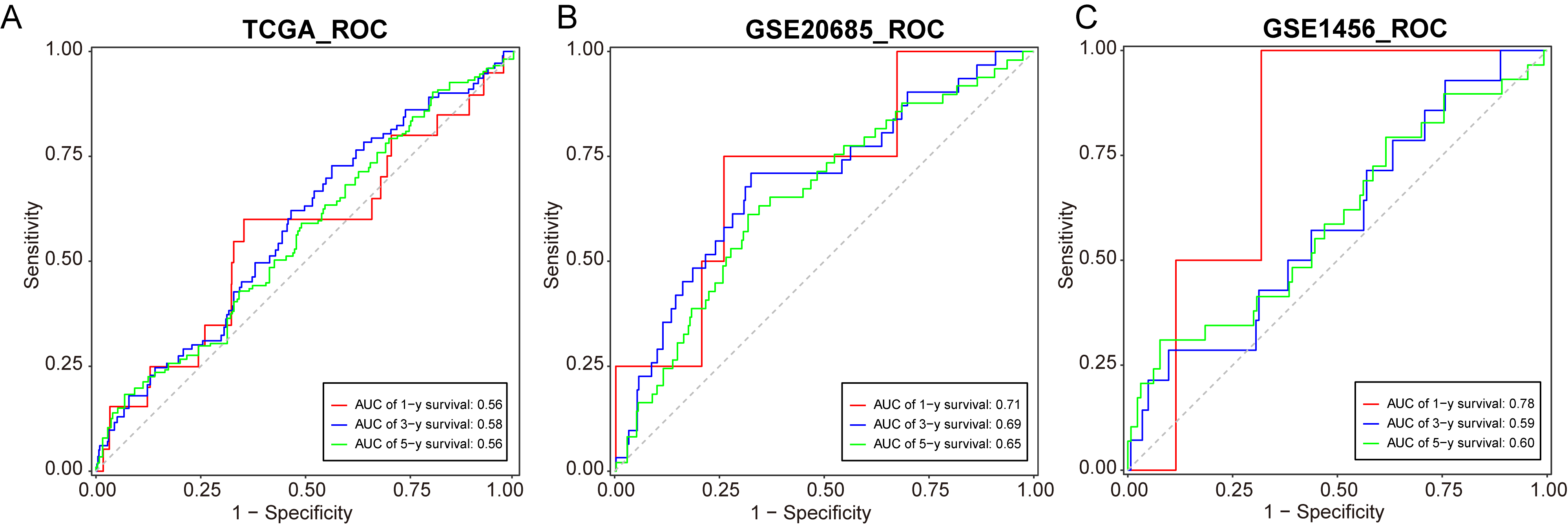

Supplement: Supplementary file 3 [file Image1.tif]

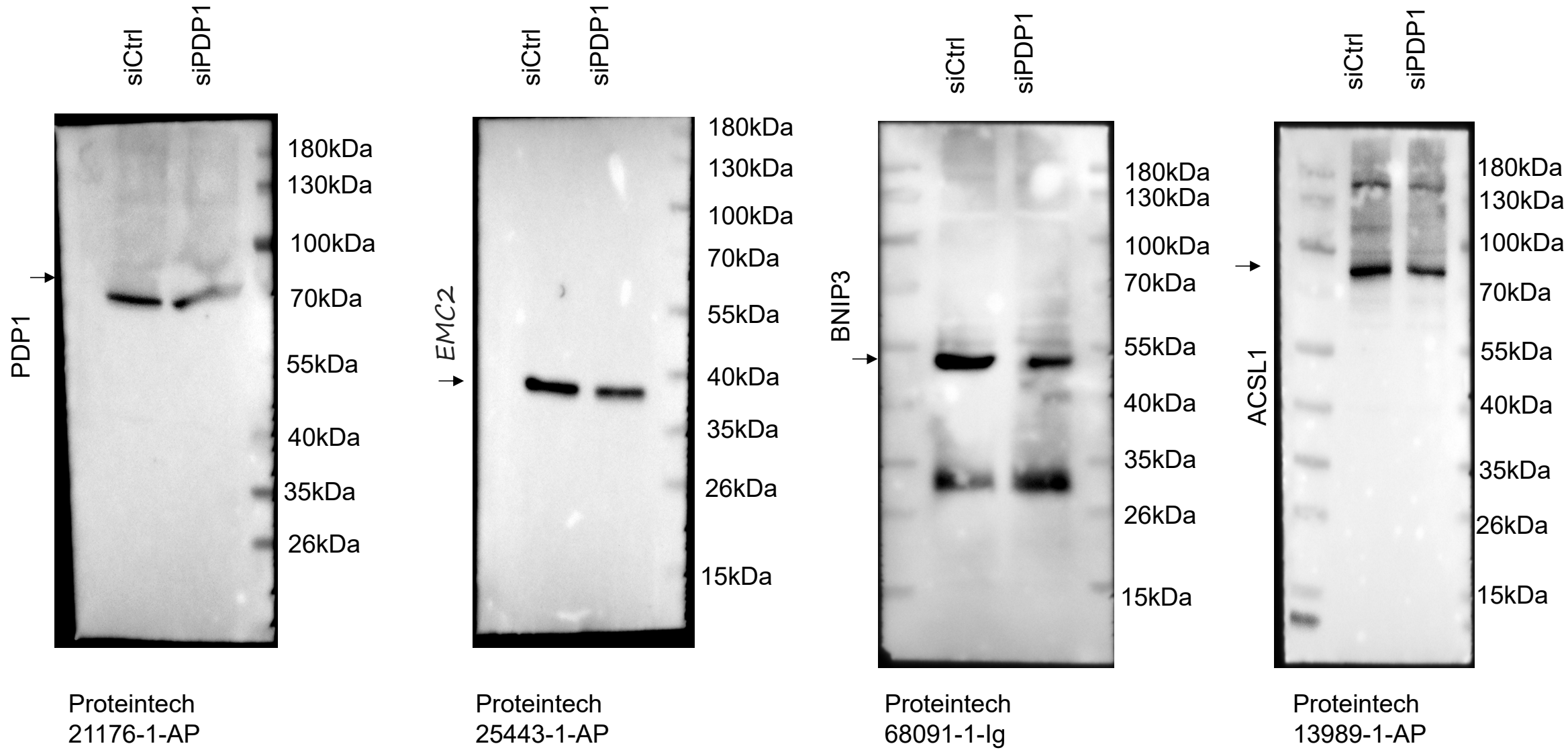

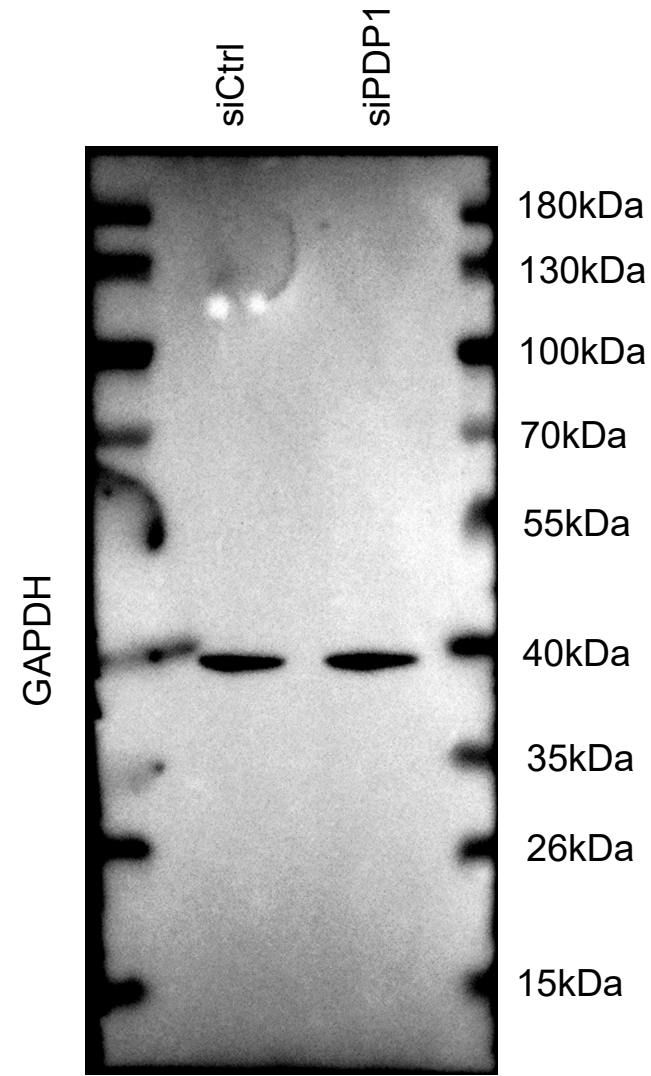

MCF7

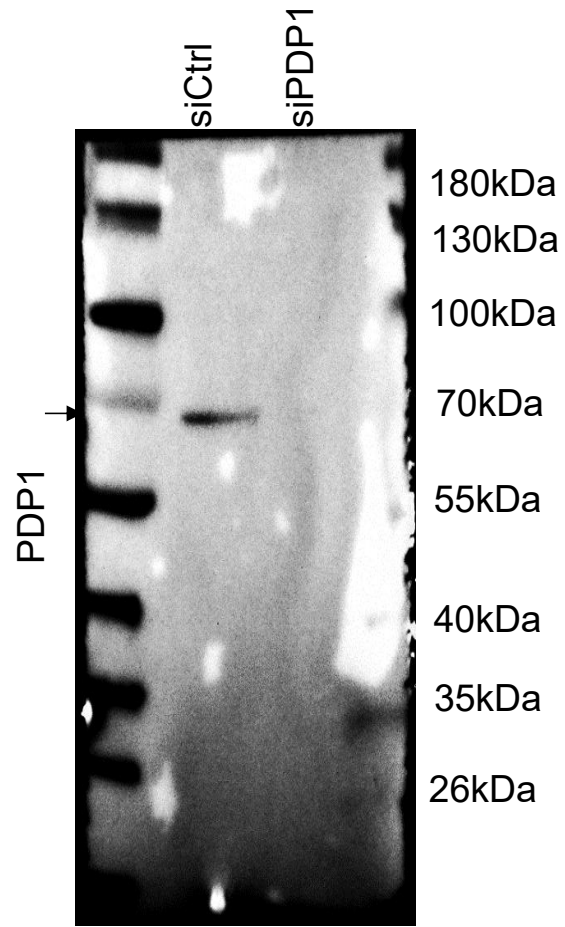

Proteintech  
21176-1-AP

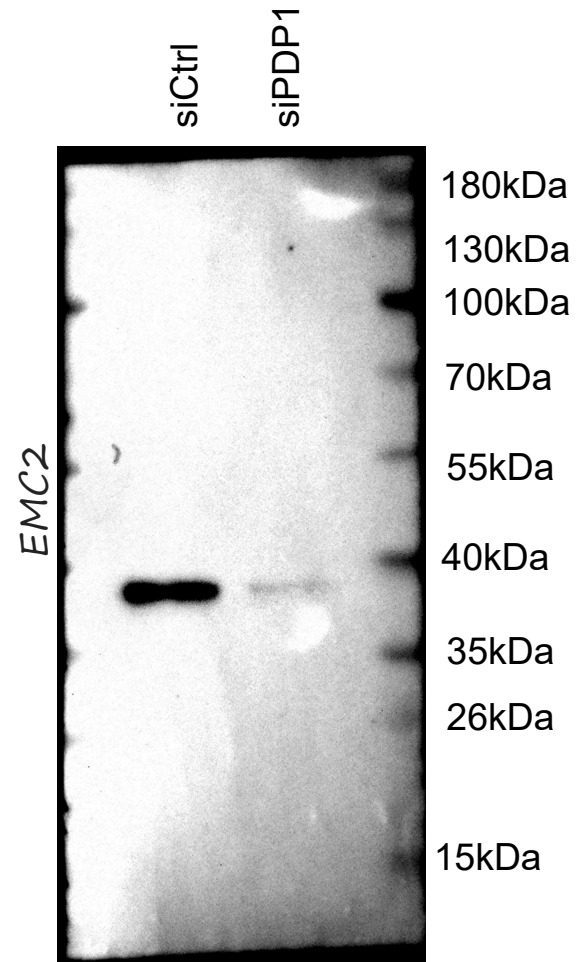

Proteintech  
25443-1-AP

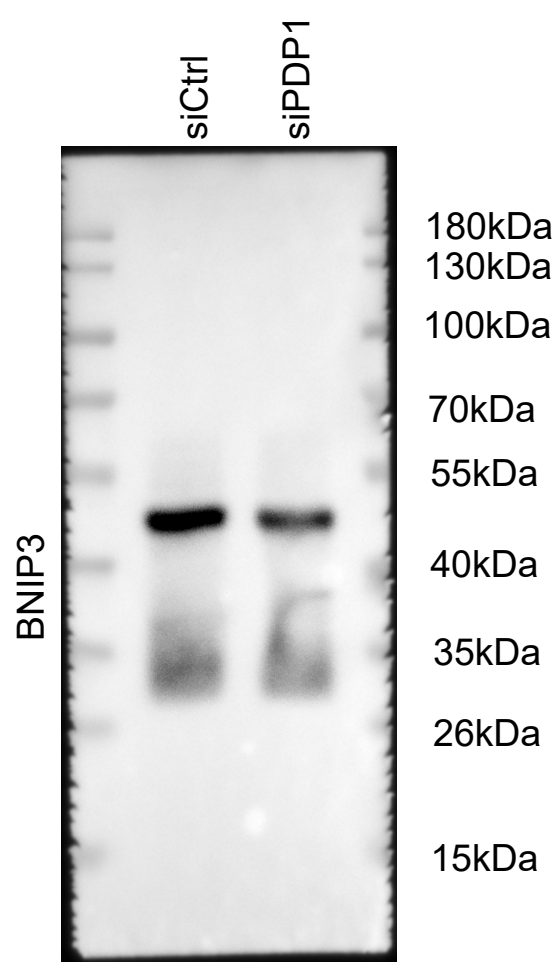

Proteintech  
68091-1-Ig

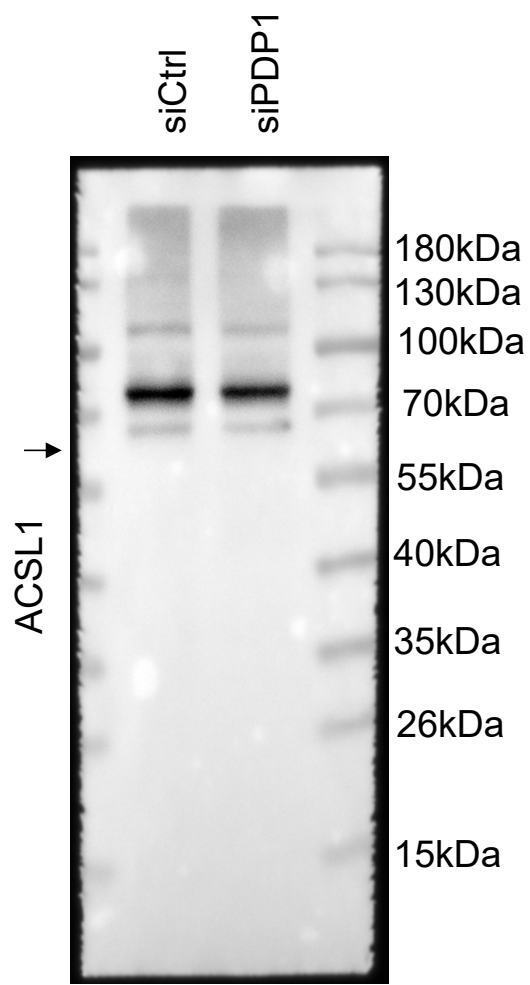

Proteintech  
13989-1-AP

MCF7

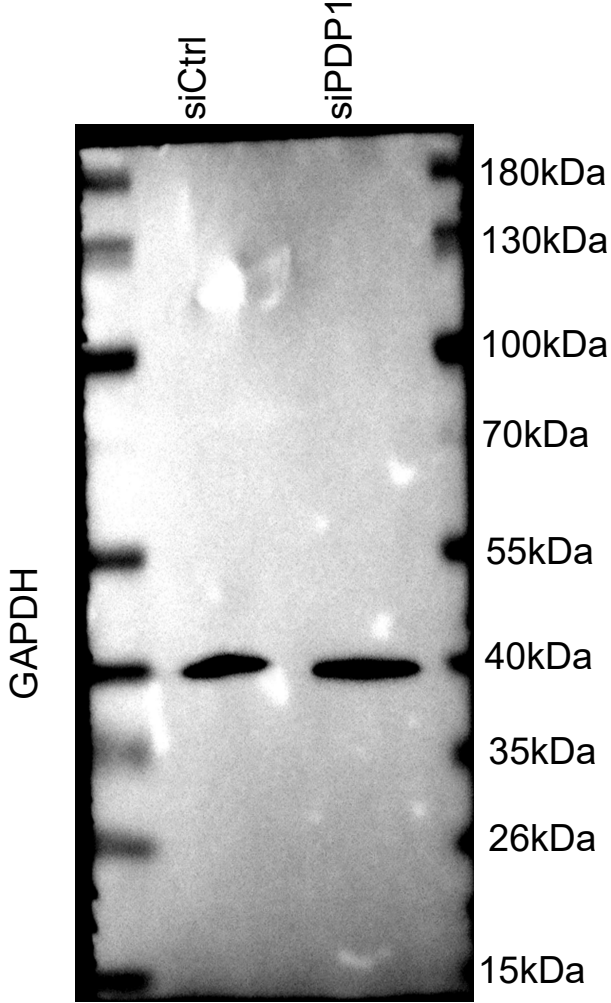

Supplement: Supplementary file 4 [file Image3.pdf]
